# Home Blood Pressure Telemonitoring and Hypertension Management in Kenya: A Feasibility Study (HBPT-K)

**DOI:** 10.5334/gh.1516

**Published:** 2026-01-14

**Authors:** Job van Steenkiste, Lilian Mbau, Helen Nguchu, Kennedy Okinda, Ruben de Neef, Bernard Samia, Daan Dohmen

**Affiliations:** 1 Faculty of Management Sciences, Open University, Valkenburgerweg 177, 6419 AT Heerlen, The Netherlands; 2 Department of Internal Medicine, Maasstad Hospital, Maasstadweg 21, 3079DZ Rotterdam, The Netherlands; 3 Department of Hospital Pharmacy, Erasmus MC University Medical Center, Dr. Molewaterplein 40, 3015 GD Rotterdam, The Netherlands; 4 Centre for Cardiovascular Prevention and Rehabilitation, Lavington, Nairobi, Kenya; 5 Department of Medicine, Mwai Kibaki Hospital-KNH Annex, Othaya-Nyeri Road, 541, 10106 Othaya, Kenya; 6 Research and Programs Department, Mwai Kibaki Hospital-KNH Annex, Othaya-Nyeri Road, 541, 10106 Othaya, Kenya; 7 Luscii Healthtech BV, Nicolaas Beetsstraat 216, 3511 HG Utrecht, The Netherlands; 8 MP Shah Hospital, Shivachi Rd, 14497, Nairobi, Kenya

**Keywords:** telemonitoring, hypertension, drug adherence, remote patient monitoring

## Abstract

**Objective:**

To determine the feasibility of home blood pressure telemonitoring (HBPT) in Kenya and explore its effects on blood pressure (BP) regulation, self-reported drug adherence, patient- and healthcare provider satisfaction, and required telemonitoring time investment.

**Methods:**

Six-month single-arm interventional feasibility study. Hundred adults with newly diagnosed or known hypertension with an office BP > 140/90 mmHg were provided with a BP machine and were enrolled in an HBPT program. Primary outcome was BP control (% BP < 140/90 mmHg) between baseline and T = 6 months (SPRINT standardized in-office blood pressure measurement). Secondary outcomes included self-reported adherence (MARS-5 scale), patient- and healthcare provider satisfaction (TUQ and MAUQ questionnaires), and efficiency (time spent processing the blood pressure telemonitoring data).

**Results:**

Between March 2024 and January 2025, 100 patients gave informed consent to participate in the study. Eighty-four patients (mean age 54, SD = 14, 73% females) completed the six-month follow-up and were included in the final analysis. Blood pressure control improved from 0% to 72% after six months (P < 0.0001). Median MARS-5 score at baseline was 25 (IQR 25–25) and remained 25 (IQR 25–25) at T = 6 months. Patient satisfaction scores were high with a median mHealth App Usability Questionnaire (MAUQ) score (range 1–7) of 7 (IQR 6.97–7) and a median Telehealth Usability Questionnaire (TUQ) (range 1–7) score of 6.95 (IQR 6.86–7). Patients participated for an average of 9.2 months in the telemonitoring program and required an E-nurse time investment of 51.7 minutes to process BP data.

**Conclusions:**

HBPT is feasible and improved BP control in a rural setting with limited time investments and high patient- and healthcare provider satisfaction rates.

**Trial Registration:**

This study is registered with the Pan African Clinical Trial registration (pactr.samrc.ac.za, trial ID: PACTR202408912454189).

## Introduction

Hypertension is the most significant risk factor for cardiovascular diseases (CVD), which are the fifth leading cause of death in Kenya (1). A nationwide survey conducted in 2015 in Kenya found 24% of Kenyans between 18 and 69 years to have hypertension (2). Only 22% of these patients were on treatment, with only 50% having a controlled blood pressure (BP) (3). Strategies leading to more effective lowering of BP are known to result in a greater reduction in cardiovascular risks (4). Lifestyle interventions like smoking cessation, reduced salt intake, and increased physical activity combined with drug therapy remain the interventions of choice in patients with hypertension. However, low drug therapy adherence is amongst the leading causes of poor BP control in adults worldwide (5). For these subjects, home blood pressure telemonitoring (HBPT) has been proposed to improve BP control and potentially increase medication and lifestyle adherence (6). HBPT involves patients measuring their BP at home (7) and these measurements are transmitted to their healthcare providers digitally. The patient is remotely monitored in a pro-active way as off-target BP values trigger alerts that allow healthcare providers to adjust treatment accordingly. On-target individuals do not trigger alerts, and the healthcare providers’ attention will therefore shift to the patient group that requires it.

Recent meta-analyses on HBPT show improved BP control, but heterogeneity amongst studies is high and data on durability is lacking (8,9). Besides the ability to remotely monitor patients, telemonitoring platforms also offer the opportunity to provide patients with educational materials (e.g., importance of medication adherence). (Automated) pro-active coaching strategies, on top of HBPT, might further enhance BP control; however, limited data is available (8). Additionally, HBPT has the potential to improve health equity by improving healthcare access (10) and can also lead to more efficient care delivery of hypertension management if offered remotely (10). This could both be relevant in areas with limited healthcare access and a relatively low healthcare provider-to-patient ratio (11) and a high smartphone penetration rate (12), like rural Kenya.

Studies on the potential effects of pro-active (active alert triggering in off-target patients) HBPT in an African population with hypertension have not been conducted, and specific data on the effects on BP control, drug adherence, and healthcare consumption are therefore not available. Given the technical novelty and complexity of HBPT in this setting, we opted to conduct a prospective feasibility study as a first step. We aimed to determine the clinical effects of HBPT and explore its potential in an adult population in rural Kenya. Specifically, we evaluated effects on BP control, efficient care delivery, patient- and health care provider satisfaction, and self-reported drug adherence.

## Methods

This study is reported in line with the 2010 CONSORT statement on feasibility trials (13). The applicable checklist can be found in Multimedia Appendix 1.

### Design

Single-arm non-randomized feasibility study.

### Setting, participants, and recruitment

This six-month feasibility study was conducted at the Mwai Kibaki Kenyatta National Hospital Annex, which is a 350-bed capacity, level six national teaching referral hospital located in Othaya Sub County, Nyeri County. We aimed to include adult patients with a new diagnosis of hypertension or with known uncontrolled hypertension. An office BP threshold of > 140/90 was chosen as the most widely reported definition of (uncontrolled) hypertension in both national and international guidelines. See Table 1 for an overview of the in- and exclusion criteria.

**Table 1:** Baseline data and demographics of the included patients.


INCLUSION CRITERIA	EXCLUSION CRITERIA

Age ≥ 18 years	Persistent atrial fibrillation as indicated in the health record

Newly diagnosed or confirmed hypertension patients with BP > 140/90 mmHg	Pregnant or planning to become pregnant during the study period

Have or have access to a smartphone with internet access throughout the study period	Severe kidney disease, defined as estimated glomerular filtration rate < 30 per 1.73 m2 or currently on renal replacement therapy (i.e., hemodialysis or peritoneal dialysis)

Able to provide written informed consent prior to participation in the study	Recent cardiovascular event (ischemic stroke, transient ischemic attack, myocardial infarction, coronary artery bypass grafting) in the past three months

	Diagnosis of dementia, psychosis, as indicated in the health record

	Life expectancy < 1 year; for instance, in terminal cancer or NYHA III or IV heart failure

	Individuals requiring BP monitor cuff size larger than 42 cm


All individuals were recruited sequentially at the study site by research assistants and/or treating physicians during regular outpatient clinic follow-up visits for hypertension.

### HBPT intervention

After giving informed consent, patients were provided with an Omron M3 Upper Arm BP monitor blood pressure machine and access to the Luscii (14) Home Blood Pressure Telemonitoring application for at least six months. The hypertension program and Luscii (14) application used in this study have been available since 2021, and have been described and evaluated in multiple studies (15,16). The program (measurement frequencies, home blood pressure monitoring technique, and threshold values) is developed in line with the latest European Society of Hypertension (ESH) guidelines (17). The Luscii (14) application consists of a smartphone or tablet application and an online healthcare provider dashboard (web page). BP data is transmitted directly to the healthcare provider dashboard via the smartphone- or tablet application. Once transmitted, the BP data is analyzed based on predefined algorithms that will subsequently generate alerts (see details below) which are only visible in the healthcare provider dashboard. In the user application, patients can review their BP data and have access to relevant educational modules and self-care documents, which, for example, emphasize the need for smoking cessation and antihypertensive drug adherence. These so-called self-care documents and educational lessons were provided to the patients both in English as well as in Swahili. See Multimedia Appendix 2 for an overview of all included educational lessons and self-care documents.

Patients measure their BP twice in the morning and twice in the evening during measurement weeks. The program consists of four acute measurement algorithms and one chronic measurement algorithm. The acute algorithms have higher frequencies of measurement weeks and stricter threshold values and are used during the initial titration phase of achieving BP control. Stable patients with BP values closer to the desired BP target values are included in the chronic algorithm and measure one week per month. See Multimedia Appendix 2 for a complete overview of the measurement algorithms.

**Figure 1:**
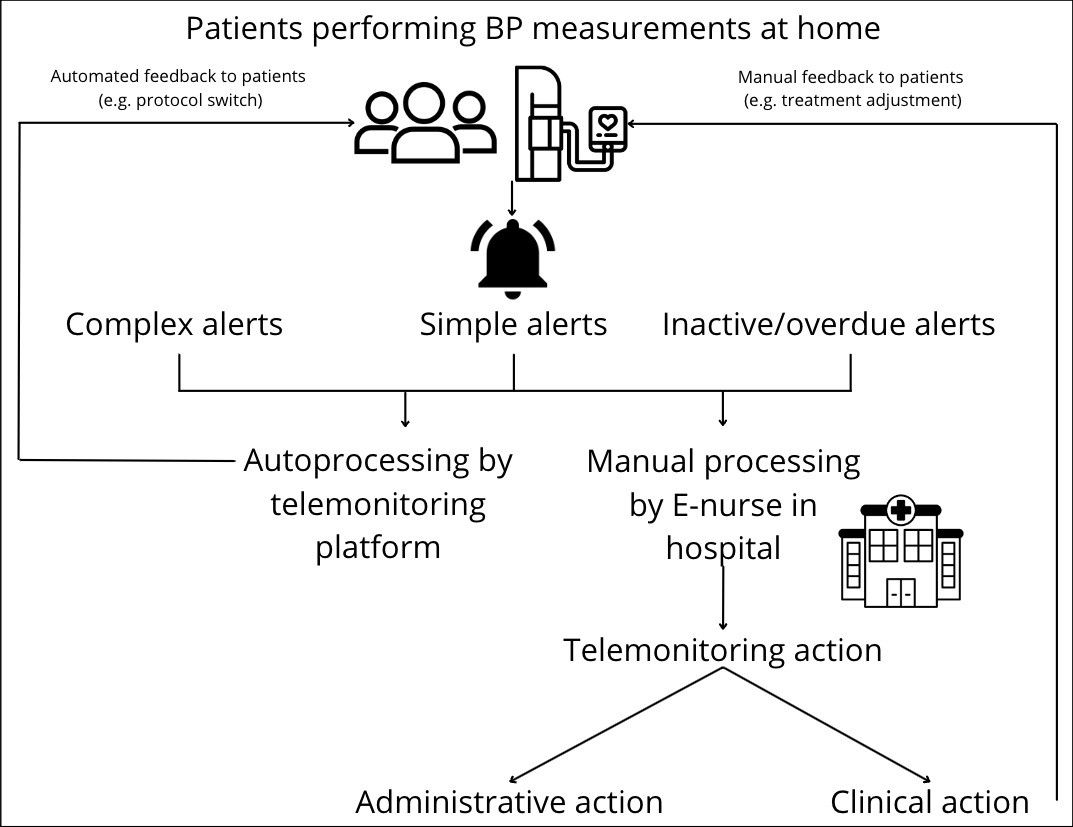
Telemonitoring organization and alert processing. Patients measure their BP at home using a validated BP machine. These measurements can trigger simple, complex, or inactive/overdue alerts. These alerts are either processed automatically by the telemonitoring platform or manually by a healthcare provider in the hospital. Feedback to the patients is provided automatically by the telemonitoring platform (e.g., a protocol switch) or manually by the E-nurse (e.g., a treatment adjustment).

The telemonitoring program generates three different types of alerts (Figure 1) following BP data transmission by the patients. First, there are simple alerts, which are triggered by a one-time measurement exceeding predefined thresholds—for example, a very high systolic BP. Second are complex alerts, which are triggered if a series of measurements are off target (e.g., multiple consecutive off-target BP measurements). In practice, these alerts are used for clinical decisions, such as drug treatment adjustments. Third are alarms that are triggered when patients either miss a single scheduled measurement (overdue alert) or when they have missed all measurements within a certain time frame (inactive alert).

Alerts can be processed automatically (e.g., a single overdue alert) by the telemonitoring platform based on automated workflows created by the responsible healthcare providers. These workflows can contain automated feedback messages that are delivered to the user automatically when the alert is processed by the monitoring platform (e.g., your BP has improved, your target has changed). Besides auto processing by the telemonitoring platform, alerts can also be processed manually (e.g., a complex alert for off-target BP) by a healthcare provider. This processing is usually done by a dedicated E-nurse (a nurse who is responsible for processing the alerts and contacting the patients if needed) and a similar set-up was used in this study. An alert is processed manually with an administrative action if no treatment adjustment is performed, and processed with a clinical action if the antihypertensive treatment is adjusted, for example.

Whether alerts need manual processing depends on choices made by healthcare providers in the telemonitoring algorithm. Prior to processing the alerts, healthcare providers can decide to contact patients to gather relevant clinical information (e.g., adherence to antihypertensive drugs of lifestyle interventions). They can also provide feedback to the patients—for example, following antihypertensive treatment adjustment. Patients were also contacted by the E-nurse following inactivity alerts.

### Outcomes and data collection

The primary outcome for this study was the difference in the proportion of BP control (% with a BP < 140/90) between baseline and T = 6 months, as measured with a SPRINT standardized in-office BP measurement (18). Secondary endpoints included:

– Differences in drug adherence as measured with the MARS-5 (19) scale between baseline and T = 6 months. Scores range from 5 to 25, where a higher MARS-5 score indicates higher self-reported adherence.
– Patient satisfaction as measured with the validated Telehealth Usability Questionnaire (TUQ) (20) and mHealth App Usability Questionnaire (MAUQ) (21) at T = 6 months. Scores range from 1 to 7, where higher TUQ and MAUQ scores indicate higher satisfaction.
– Healthcare provider satisfaction as measured with the validated mHealth App Usability Questionnaire (MAUQ) at T = 6 months. Scores range from 1 to 7, where a higher MAUQ score indicates higher satisfaction.

Additionally, at baseline, we collected demographic data (age, sex, medical history, current use of antihypertensive drugs, and smoking status). To estimate the feasibility in terms of using the HBPT program and its potential workload, we extracted telemonitoring data after the final study participant completed the T = 6-month visit. This data included the total telemonitoring participation (active days in the program), adherence to the measurement schedules, number of alerts, and data on the telemonitoring alert processing, including the time spent processing alerts. To calculate the time spent processing alerts, we assumed a workload of five minutes for a digital message, five minutes for a phone call, 10 minutes for a clinical action, and two minutes for an administrative action. We also extracted the number of physical consultations during the study period (excluding the study visits).

### Sample size

We estimated the level of BP control to increase, based on available HBPT studies conducted outside the African continent, by 20% from 0% at baseline in our study population (22). For a McNemar test to detect a 20% difference between marginal proportions at T = 6 months with a power of 80% and a two-sided significance of 5%, at least 42 individuals would need to be recruited. As this is a feasibility study and relevant data on potential dropouts and potential effects on BP control in this specific setting and population is not available, we opted for a more pragmatic approach and decided to recruit 100 study participants.

### Statistical analysis

All statistical analyses were performed following the per-protocol principle and data was only used from patients that completed the T = 6-month follow-up. Descriptive statistics were used for the demographic data. Data was reported as proportions (%), means (standard deviation), or medians (interquartile range). For the primary outcome, a McNemar test for proportions was used, and to compare the average BP (absolute systolic and diastolic values) between baseline and T = 6 months, a paired T-test was used. All statistical analyses were performed using Microsoft Excel (23) or R (24).

## Results

### Study participants and baseline data

**Figure 2:**
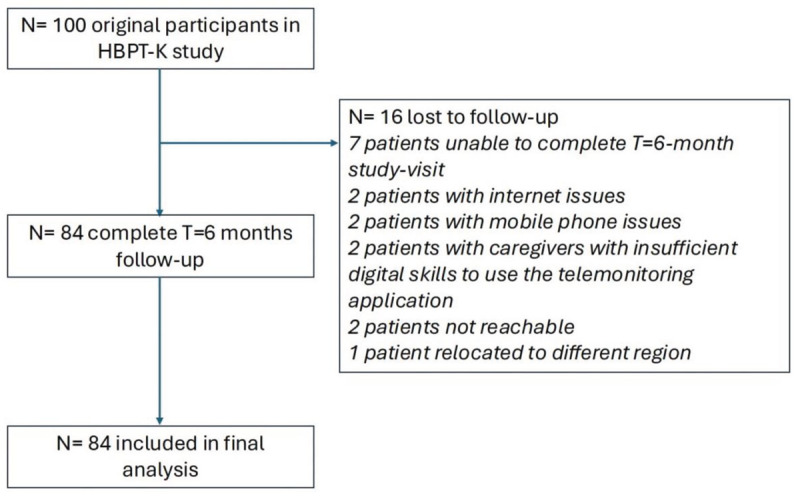
Study flowchart.

Between March 2024 and January 2025, a total of 100 patients gave informed consent to participate in the study. Out of these, 16 were lost to follow-up during the study period and therefore, a total of 84 participants were included in the final analysis. See Figure 2 for the study flowchart.

The mean age was 54 (SD = 14) and 73% were females. All patients had a known diagnosis of hypertension and 6% also reported having diabetes. Only two (2%) of the included patients reported being active smokers. See Table 2 for a complete overview of the baseline demographic data.

**Table 2:** Baseline data and demographics of the included patients.


Total number of study participants	84

Age, mean (SD)	54 (14)

Female, n (%)	61 (73%)

Hypertension, n (%)	84 (100%)

Diabetes, n (%)	5 (6%)

Smoking, n (%)	2 (2%)


### Blood pressure control, antihypertensive drug use, and consultations

At baseline, 0% of the included patients had a BP < 140/90 mmHg as measured using the SPRINT standardized in-office BP measurements (18). This increased to 72% after six months (P < 0.0001) (Table 3). Absolute BP values improved from 157 mmHg (SD 18) systolic to 128 mmHg (SD 12), and 97 mmHg (SD 8) to 82 mmHg (SD 12) after six months (p < 0.0001 for both systolic and diastolic BPs). The median number of antihypertensives used increased from 2 (IQR 2–3) to 3 (IQR 2–3) during the study period and the median number of physical consultations (excluding study visits) was 1 (IQR 0–2).

**Table 3:** Blood pressure data, antihypertensive drug use, and consultations.


% of patients with BP < 140/90 mmHg at baseline	0%	

% of patients with BP < 140/90 mmHg at six months	72%	P < 0.0001*

Baseline systolic BP mmHg (mean, SD)	157 (18)	

T = 6 months systolic BP mmHg (mean, SD)	128 (12)	P < 0.0001**

Baseline diastolic BP mmHg (mean, SD)	97 (8)	

T = 6 months diastolic BP mmHg (mean, SD)	82 (12)	P < 0.0001**

Number of antihypertensives baseline (median, IQR)	2 (2–3)	

Number of antihypertensives six months (median, IQR)	3 (2–3)	

Number of physical consultations during study period (median, IQR), not including baseline and T = 6-month study visit	1 (0–2)	


* = McNemar test for proportions; ** = paired T-test.

### Drug adherence

MARS-5 questionnaires were completed by 99% and 88% of the study participants at baseline and six months, respectively. Median MARS-5 scores at baseline were 25 (IQR 25–25) and remained 25 (IQR 25–25) at T = 6 month follow-up.

### Patient- and healthcare provider satisfaction

Both TUQ and MAUQ questionnaires were completed by 89% of the study participants. Overall patient satisfaction scores were high with a median MAUQ of 7 (IQR 6.97–7) and a median TUQ score of 6.95 (IQR 6.86–7). Four healthcare providers completed the MAUQ questionnaire for healthcare providers and satisfaction was high with a median MAUQ of 7 (IQR 6.05–7).

### Telemonitoring data

See Table 4 for a complete overview of the telemonitoring data. Telemonitoring data were extracted after the final patient completed the T = 6 month follow-up. Therefore, the total average duration of the telemonitoring program was 276 days (9.2 months). Each individual patient transmitted an average of 114 BP measurements during the study period. These measurements triggered a total of 1677 alerts, of which the majority were simple alerts (930, 55%). The HBPT platform automatically processed 35% (579) of all alerts and 65% were processed manually (1098). The total time investment based on the average alert processing for each individual patient was 51.7 minutes over 9.2 months.

**Table 4:** Telemonitoring data.


**Telemonitoring participation**	

Total average days active in the HBPT program in days (months)	276 (9.2)

Total individual measurements (heart rate, systolic BP, diastolic BP)	28785

Total combined measurements (heart rate, systolic BP, diastolic BP combined in one single measurement)	9595

Total combined measurements per participating patient	114

Average measurement schedule adherence*	22%

**Telemonitoring alerts**	

Total alerts	1677

Simple alerts	930 (55%)

Complex alerts	747 (45%)

Total alerts per patient	20

**Telemonitoring alert processing**	

Alerts auto processed by telemonitoring platform (% total alerts)	579 (35%)

Manual alerts processed (% total alerts)	1098 (65%)

*Administrative action (% total manually processed alerts)*	612 (56%)

*Contacted via phone (% total manually processed alerts)*	339 (31%)

*Contacted via message (% total manually processed alerts)*	9 (1%)

*Clinical action (% total manually processed alerts)*	138 (12%)

**Telemonitoring time investment**	

Phone (5 min), minutes, average per patient	20.2

Message (5 min), minutes, average per patient	0.5

Clinical action (10 min), minutes, average per patient	16.4

Administrative action (2 min), minutes, average per patient	14.6

Total average time investment (minutes) per patient during 9.2 months of telemonitoring	**51.7**


*515 measurements were expected based on an average bi-weekly measurement week with 28 combined measurements for a period of 9.2 months. Adherence was calculated by dividing the average combined number of measurements by the expected number of measurements (114/515).

## Discussion

This feasibility study on the effects of HBPT in an adult population with uncontrolled hypertension in Kenya showed a significant improvement in BP as 72% of the included individuals had a BP < 140/90 mmHg following a six-month period of HBPT. Besides the positive effects on BP control, we found patient- and healthcare provider satisfaction to be high. HBPT also appeared to be feasible in terms of required healthcare provider (E-nurse) time investment, with an average time consumption of 51.7 minutes for the telemonitoring over 9.2 months. Additionally, HBPT was effective in optimizing care delivery by improving patient outcomes without increasing the number of physical visits. This finding is especially relevant in underserved areas. Finally, we were unable to show improvement in drug adherence after using HBPT; however, this was likely a result from selection bias given the very high self-reported adherence at baseline (25).

### Exploring the effects of HBPT

We believe the positive effects of HBPT on hypertension control rates to be multi-factorial.

HBPT is a complex and multidimensional intervention and the combination of measurements, remote monitoring, and digital lifestyle interventions can all contribute individually to improved clinical outcomes (8,9). Given the small size and design of our study, we were unable to specifically point towards a specific HBPT effect. As existing studies (8,9) have shown, the identified positive effect on BP control is most likely the result of a combination of increased healthcare provider disease insight and awareness, reduced physician inertia (26), increased patient disease insight, increased drug adherence, and lifestyle modification (22). This was also partly underscored by an increased number of antihypertensive drugs, which could indicate that the HBPT program addressed physician inertia, which is known to be an important cause of ongoing uncontrolled hypertension (26). These findings are consistent with other studies that demonstrated HBPT to be associated with improved systolic and diastolic blood pressure, an increase in the number of antihypertensive medications prescribed, but with unchanged drug adherence and clinic visits as compared to usual care (11,27).

### Feasibility of HBPT in underserved areas

Implementing a digital healthcare system like HBPT in an underserved area appeared to be feasible based on our study findings and could thereby improve healthcare access, which is in line with similar results from HBPT studies in underserved areas outside of the African continent (10). This conclusion was further supported by a low drop-out rate and fewer patients experiencing connectivity and technology-related issues during the study period. The involved healthcare providers also did not experience any implementation or technical issues with data transmission or the telemonitoring process itself. Additionally, the overall HBPT set-up and telemonitoring infrastructure could be further improved: for example, with the use of a dedicated remote patient monitoring center (6). Further automation of the alerts—for example, by using artificial intelligence—would also allow to further scale-up the current system. Using multiple E-nurses in a remote patient monitoring center would also improve the vulnerability of the required human intervention in managing the alerts, as we only used a single E-nurse in this study. On the other hand, we did experience a relatively low adherence to the measurement schedule, which might negatively affect long-term sustainability. The introduction of specific measures like more flexible and shorter measurement schedules and the introduction of an element of gamification with rewards could help improve this.

### Study limitations

The small sample size and design need to be considered when interpreting results of this study. Specifically, the lack of a control group affects the interpretation of the clinical effect of the HBPT intervention as compared to usual care without HBPT. Additionally, to estimate drug adherence, we used a self-reporting adherence tool (MARS-5). Self-reports tend to overestimate adherence compared to more objective assessment methods like measuring antihypertensive drug concentrations in blood or urine samples. These advanced measurement methods, however, were not available given the study setting.

Additionally, we did not incorporate any cost-effective or cost-utility analysis. Patients were provided with a BP machine and the HBPT app was provided free of cost. True long-term adherence and feasibility might depend on the availability of the BP machines, the funding of the HBPT application, and the continuous availability of mobile internet to transmit BP values. Also, we used a cut-off of 140/90 mmHg for all studies irrespective of cardiovascular risk, which might be inappropriate for certain patients. Further studies should therefore include a specific telemonitoring program with a 135/85 mmHg target where applicable. Finally, the adherence to the measurement schedule was low, which could have significantly impacted the effects of the HBPT intervention on multiple outcome measures.

## Conclusions

Our feasibility study on HBPT in Kenya showed a large potential for HBPT in improving BP control in a rural setting with minimal time investments, few necessary physical consultations, and high patient- and healthcare provider satisfaction rates. Future studies should focus on long-term outcomes of HBPT in a similar setting and on exploring the underlying mechanisms by which HBPT leads to improved clinical outcomes. Future studies should also include direct (blood or urine) antihypertensive drug adherence measurements to identify subgroups with low adherence and isolate the potential effect of HBPT on drug adherence. In addition, there is need to assess the cost-effectiveness of HBPT in resource-limited and rural settings.

## Data Accessibility Statement

Data reported in this study is available upon reasonable request to the corresponding author.

## Additional Files

The additional files for this article can be found as follows:

10.5334/gh.1516.s1 Multimedia Appendix 1. CONSORT 2010 checklist of information to include when reporting a pilot or feasibility trial.

10.5334/gh.1516.s2 Multimedia Appendix 2. Multimedia appendix 2: HBPT measurement algorithms, self-care documents and educational lessons.

## Abbreviations

BP: Blood pressure

CVD: Cardiovascular diseases

ESH: European Society of Hypertension

GCP: Good Clinical Practice

HBPT: Home blood pressure telemonitoring
