# Supplementary material for: Home Blood Pressure Telemonitoring and Hypertension Management in Kenya: A Feasibility Study (HBPT-K)
Source: Glob Heart. 2026 Jan 14;21(1):1. doi: 10.5334/gh.1516 (PMC12802096; doi:10.5334/gh.1516)
Supplement: Multimedia Appendix 2. — Multimedia appendix 2: HBPT measurement algorithms, self-care documents and educational lessons. [file gh-21-1-1516-s2.pdf]

Multimedia appendix 2: HBPT measurement algorithms, self-care documents and educational lessons.

Algorithm	Measurement frequency	Simple alerts	Complex alerts	Monitoring frequency
180/110	Daily (twice morning/twice evening)	RR sys >180 of dias >110<br>RR <100 sys<br>HF <45<br>HF >120	4 or more readings above/below target in last 48 hours (systolic and diastolic blood pressure, heart rate)	Monday to Friday
170/105	Daily (twice morning/twice evening)	RR sys >180 of dias >110<br>RR <100 sys<br>HF <45<br>HF >120	4 or more readings above/below target in last 48 hours (systolic and diastolic blood pressure, heart rate)	Monday to Friday
160/100	One whole week every fortnight (twice morning/twice evening)	RR sys >170 of dias >105<br>RR <100 sys<br>HF <45<br>HF >120	8 or more readings above/below target in last 7 days (systolic and diastolic blood pressure, heart rate)	Monday to Friday
150/95	One whole week every fortnight (twice morning/twice evening)	RR sys >170 of dias >105<br>RR <100 sys<br>HF <45<br>HF >120	8 or more readings above/below target in last 7 days (systolic and diastolic blood pressure, heart rate)<br>>1-week average BP within range – move to next protocol’	Monday to Friday
140/90	One whole week every month (twice morning/twice evening)	RR sys >170 of dias >105<br>RR <100 sys<br>HF <45<br>HF >120	14 or more readings above/below target in last 7 days (systolic and diastolic blood pressure, heart rate)	Monday to Friday
Algorithm switch alert (blood pressure on target)			1. End of the measurement week: half of all measurements (with at least 14 measurements performed) are within range -> automated switch to next Algorithm	Not applicable

**Self-care documents**	**Educational lessons**
**1.** What is home blood pressure monitoring?	**1.** How does home blood pressure monitoring work?
**2.** What should I do in case of a heat wave or a fever?	**2.** How to correctly measure your blood pressure at home
**3.** What should I do when vomiting or having diarrhoea?	**3.** What is hypertension?
**4.** I'm going for a holiday, how can I temporarily suspend my measurments	**4.** Hypertension and lifestyle, a healthy diet, salt intake, physical activity, a healthy weight, smoking cessation, licorice
**5.** What should I do when I deviated from my measurement schedule?	**5.** Antihypertensive drugs
**6.** What should I do when I forgot to take my antihypertensive drugs?	**6.** Stress and hypertension
**7.** I'm having symptoms that could be related to my blood pressure, what should I do?	
**8.** Can I still enter my blood pressure measurements from yesterday?	
**9.** How can I manually enter additional blood pressure measurements?	
